# Expression profile of mucins (MUC2, MUC5AC and MUC6) in *Helicobacter pylori *infected pre-neoplastic and neoplastic human gastric epithelium

**DOI:** 10.1186/1476-4598-5-10

**Published:** 2006-03-19

**Authors:** Subramani Durai Babu, Venkataraman Jayanthi, Niranjali Devaraj, Celso A Reis, Halagowder Devaraj

**Affiliations:** 1 Unit of Biochemistry, Department of Zoology, University of Madras, Guindy, Chennai, India; 2 Department of Gastroenerology, Stanley Medical College and Hospital, Chennai, India; 3 Department of Biochemistry, University of Madras, Guindy Campus, Chennai, India; 4 IPATIMUP – Institute of Molecular Pathology and Immunology of the University of Porto, Portugal

## Abstract

**Background:**

*Helicobacter pylori *(*H. pylori*) causes gastritis and intestinal metaplasia (IM) that may evolve to gastric carcinoma. The objective of this study was to compare the profile of mucins in the progressive stages of *H. pylori *infected pre-neoplastic and neoplastic human gastric epithelium. We used a panel of monoclonal antibodies with well-defined specificities of MUC2, MUC5AC and MUC6 to characterize the expression pattern of mucins by immunohistochemistry.

**Methods:**

RUT and ELISA were down for *H. pylori *confirmation. Human gastric biopsy sections were stained using immunohistochemistry with MUC2, MUC5AC and MUC6 antibodies.

**Results:**

MUC5AC was expressed in the superficial epithelium and the upper part of the gastric pits. MUC6 expression was detected in the lower part of the gastric glands. MUC2 was expressed in intestinal metaplasia, mostly in goblet cells. The mucin expression profile in the progressive stages of *H. pylori *infected human gastric epithelium allows the identification of intestinal metaplasia, which is characterized by a decreased expression of the gastric mucins (MUC5AC and MUC6) and *de novo *expression of MUC2.

**Conclusion:**

In conclusion, our results suggest that there is altered expression of MUC5AC and MUC6 together with the aberrant expression of MUC2 in intestinal metaplasia, during the process of gastric carcinogenesis. The present study indicates that the MUC2 mucin expression pattern is a reliable marker of intestinal metaplasia, which appears in the context of *H. pylori *infected individuals.

## Background

*Helicobacter pylori *colonizes human gastric mucosa and causes gastritis and intestinal metaplasia (IM), which may evolve towards gastric carcinoma [1-3]. *H. pylori *infection was established as a type I human carcinogen in IARC [4]; it was recently shown that the bacteria are also capable of inducing gastritis, IM, and gastric carcinoma in Mongolian gerbils [5-8]. In humans, *H. pylori *also colonizes the duodenal mucosa whenever there is gastric metaplasia [9]. *H. pylori *infection in human is therefore dependent on the gastric microenvironment, which is determined to a large extent by the mucin and carbohydrate composition of the gastric mucin layer.

Mucins are heavily glycosylated glycoproteins that are the major components of the mucous viscous gel covering the surface epithelial tissues [10]. To date, nine distinct epithelial mucin genes (MUC1, 2, 3, 4, 5AC, 5B, 6, 7, and 8) have been identified [11-21]. *In situ *hybridization and immunohistochemical studies have shown that these mucins are differentially expressed in epithelia with cell type specificity [21-24]. The normal gastric mucosa shows cell types specific expression of MUC1, MUC5AC, and MUC6, with first two mucins found in the superficial epithelium and MUC6 in the deep glands [22,25,26]. MUC6 is mainly expressed in gastric mucosa. The normal gastric mucosa does not express MUC2 [25,27].

Changes in the expression levels and glycosylation patterns of mucins have been associated with several diseases, including carcinomas [27-31]. In gastric cancer, alterations in mucin polypeptide have been reported: loss of expression of MUC5AC [23,26,32], increased mucin heterogenesity [23]. These observations suggest that mucin alterations can be regarded as "molecular" markers of malignant transformation of gastric mucosa. Further more, a recently published investigation strongly suggested MUC5AC as a putative *H. pylari *receptor [33,34].

In this present study, we have characterized the pattern of mucins expression in the progressive stages of *H. pylori *infected human gastric carcinoma. We have used a comprehensive panel of monoclonal antibodies, with well-characterized specificities directed to MUC2, MUC5AC and MUC6 mucins.

## Results

### Expression pattern of MUC2, MUC5AC and MUC6 mucins in normal gastric mucosa

**Figure 1:**
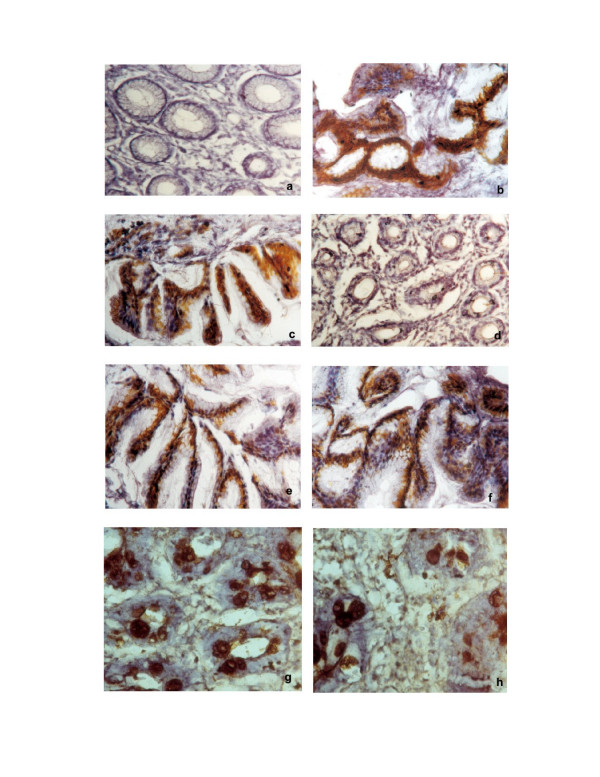
**Immunohistochemical staining of MUC2, MUC5AC and MUC6 mucins in *H. pylori *infected patients**. **a**. MUC2 staining of normal gastric mucosa (Original magnification ×100). **b**. MUC5AC staining of normal gastric mucosa (Original magnification ×100). **c**. MUC6 staining of normal gastric mucosa (Original magnification ×100). **d**. MUC2 staining of Atrophic gastritis (Original magnification ×100). **e**. MUC5AC staining of Atrophic gastritis (Original magnification ×100). **f**. MUC6 staining of Atrophic gastritis (Original magnification ×100). **g**. MUC2 staining of Intestinal metaplasia (Original magnification ×100). **h**. MUC5AC staining of Intestinal metaplasia (Original magnification ×100).

MUC5AC was highly expressed in foveolar epithelium and mucous neck cells of antrum (> 75%, Fig. 1b; Fig. 3b; Table 2). Expression of MUC6 was detected in the glands of the antrum (> 75%, Fig. 1c; Fig. 3c; Table 3) and MUC2 was not detected in normal gastric mucosa (Fig. 1a; Fig. 3a; Table 2).

**Figure 2:**
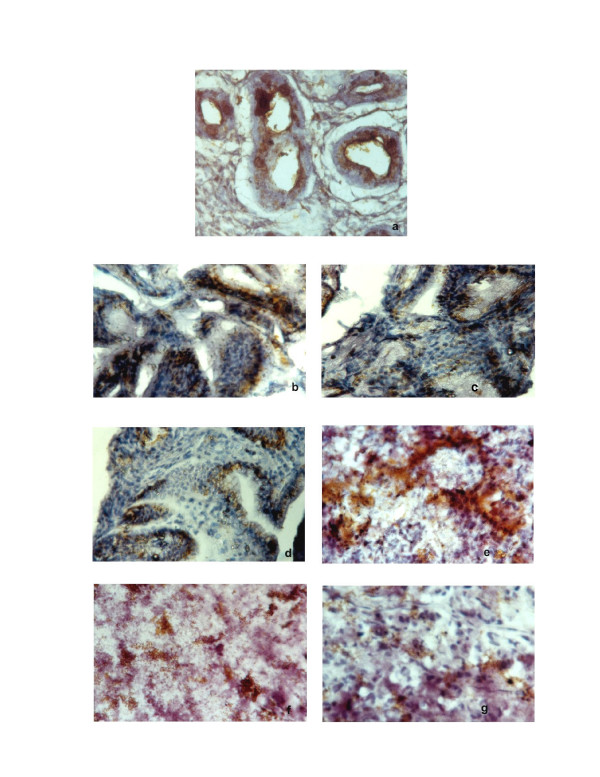
**Immunohistochemical staining of MUC2, MUC5AC and MUC6 mucins in *H. pylori *infected patients**. **a**. MUC6 staining of Intestinal metaplasia (Original magnification ×100). **b**. MUC2 staining of dysplasia (Original magnification ×100). **c**. MUC5AC staining of dysplasia (Original magnification ×100). **d**. MUC6 staining of dysplasia (Original magnification ×100). **e**. MUC2 staining of carcinoma (Original magnification ×100). **f**. MUC5AC staining of carcinoma (Original magnification ×100). **g**. MUC6 staining of carcinoma (Original magnification ×100).

**Figure 3:**
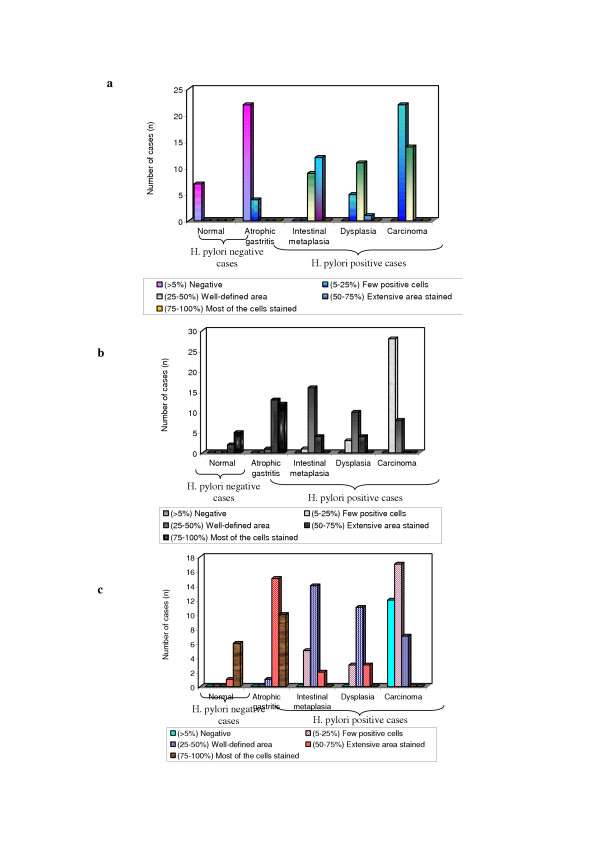
**Expression pattern of mucins (MUC2, MUC5AC and MUC6) in *H. pylori *infected pre-neoplastic and neoplastic human gastric epithelium**. a. MUC2 expression in *H. pylori *infected pre-neoplastic and neoplastic human gastric epithelium. b. MUC5AC expression in *H. pylori *infected pre-neoplastic and neoplastic human gastric epithelium. c. MUC6 expression in *H. pylori *infected pre-neoplastic and neoplastic human gastric epithelium.

**Table 1:** Specificities, origins and dilutions of the monoclonal antibodies.

**Monoclonal antibody**	**Specificity**	**Reference**	**Dilution**
PMH1	MUC2	Reis *et al *[11]	Undiluted
CLH2	MUC5AC	Reis *et al *[12]	1:2
CLH5	MUC6	Reis *et al *[10]	1:2

**Table 2:** Expression of mucins (MUC2, MUCSAC and MUC6) in *H. pylori *infected pre-neoplastic and neoplastic human gastric epithelium

**Stages**	H. pylori		**MUC2 Expression**					**MUC5AC Expression**					**MUC6 Expression**				
	
	**N**	**P**	**-**	**+**	**++**	**+++**	**++++**	**-**	**+**	**++**	**+++**	**++++**	**-**	**+**	**++**	**+++**	**++++**
Normal (n = 7)	7	-	n = 7 (100%)	-	-	-	-	-	-	-	n = 2 (28.6%)	n = 5 (71.4%)	-	-	-	1 (14.3%)	6 (85.7%)
Atrophic gastritis (n = 26)	-	26	n = 22 (84.6%)	n = 4 (15.4%)	-	-	-	-	-	1 (38%)	13 (50%)	12 (46.2%)	-	-	1 (3.8%)	15 (57.7%)	10 (38.5%)
Intestinal metaplasia (n = 21)	-	21	-	-	n = 9 (42.9%)	n = 12 (57.1%)**	-	-	1 (4.8%)	16 (76.2%)**	4 (19%)	-	-	5 (23.8%)	14 (66.7%)**	2 (9.5%)	-
Dysplasia (n = 17)	-	17	-	n = 5 (29.4%)	n = 11 (64.7%)	n = 1 (5.9%)	-	-	3 (17.6%)	10 (58.8%)	4 (23.5%)	-	-	3 (17.6%)	11 (64.7%)	3 (17.3%)	-
Carcinoma (n = 36)	-	36	-	22 (61.1%)	14 (38.9%)	-	-	-	28 (77.8%)	8 (22.2%)	-	-	12 (33.3%)	17 (47.2%)	7 (19.4%)	-	-

N – negative - (> 5%) negative

P – positive + (5–25%) few positive cells

n – No. of cases ++ (25–50%) well-defined area

+++ (50–75%) extensive area stained

++++ (75–100%) most of the cells stained

**p < 0.00, *p < 0.05 (Significant difference in expression)

### Immunodetection of mucins in atrophic gastritis

There was expression of MUC5AC in all the cases of columnar cells (Fig. 1e; Fig. 3b; Table 2). The percentage of stained cells varied from case to case, in most cases, there was a trend for higher number of cells expressing MUC5AC (>75%) in the superficial part of the metaplastic glands than in deep part. MUC6 was detected in columnar cells (>50%; Fig. 1f; Fig. 3c; Table 2) and MUC2 was not detected in atrophic gastritis (Fig. 1d).

### Immunodetection of mucins in intestinal metaplasia

There was decreased level expression of MUC5AC and MUC6 in all the cases (>50%), both in goblet and in columnar cells (Fig. 1h, Fig. 2a; Fig. 3b, Fig. 3c; Table 2). The percentage of stained cells varied from case to case, *de novo *expression of intestinal mucin MUC2 was observed (>75%), and its expression displayed a diffuse cytoplasmic pattern in goblet cells (Fig. 1g; Fig. 3a; Table 2).

### Immunodetection of mucins in dysplasia

There was decreased level expression of MUC5AC and MUC6, than intestinal metaplasia (>50%). MUC5AC and MUC6 displayed a diffuse cytoplasmic expression pattern in columnar cells (Fig. 2c; Fig. 2d; Fig. 3d; Fig. 3; Table 2). MUC2 expression displayed a diffuse cytoplasmic pattern in goblets cells (>50%, Fig. 2b; Fig 3a; Table 2).

### Immunodetection of mucins in carcinoma

MUC5AC and MUC6 expression were frequently observed in early carcinoma (>50%) than advanced carcinomas (>25%). In advanced carcinomas, we observed a trend for decreased immunoreactivity in deep areas than in the superficial areas. MUC5AC and MUC6 expressions were decreased in advanced carcinomas (>25%; Fig. 3a; Fig. 3b; Fig. 3c; Fig. 2e, Fig. 2f, Fig. 2g; Table 2).

## Discussion

In this present study, we have shown that the pattern of mucins expression in the progressive stages of *H. pylori *infected human gastric epithelium. Mucins are expressed with a cell- and tissue-specific pattern in normal tissue. Alterations of the expression pattern of mucins have been described in carcinomas as well as in their precursor lesions; in the latter, altered mucin carbohydrate and peptide moieties may constitute molecular markers of increased risk of malignant transformation [27,35,36,38-40].

In agreement with previous studies reporting the distribution of mucins in normal stomach, we found MUC5AC is highly expressed in foveolar cells of antrum, as reported [26,27,32,37-39]. We also found that MUC6 is expressed in mucous cells of the neck zone in pyloric glands of the antrum with similar pattern to that described previously [22,23,47]. The expression of MUC2 was usually not detected in normal gastric mucosa as described in the previous reports [25,27,40].

Intestinal metaplasia is one of the lesions identified in the cascade of event that precedes the development of gastric carcinoma [41]. Intestinal metaplasia comprises the replacement of the gastric mucosa by an epithelium that resembles histologically the intestinal mucosa. Altered mucin expression patterns have been observed in *H. pylori *infected intestinal metaplasia, including under expression of MUC5AC and MUC6 and *de novo *expression of MUC2 mostly in goblet cells [30,48]. These results support the assumption that the intestinal metaplasia does represent a differentiation of the mucosa toward a intestinal phenotype [42,43].

The decreased expression of MUC5AC and MUC6 mucin in gastric carcinomas were confirmed in the present study. The expression of MUC5AC in early gastric carcinoma of the present series, regardless of the histological type of the tumors, contrasts with the decreased level expression of MUC5AC immunoreactivity in almost half of the advanced carcinomas which suggest that all gastric carcinomas retain at least some cells with a gastric phenotype during the first steps of neoplastic development (26). This assumption is further supported by the decreased immunoreactivity in deep areas compared with superficial areas of the advanced carcinomas. These results are in agreement with previous results showing that gastric cancer progression is accompanied by alteration of the expression of several mucin genes [14]. Finally, we observed that the intestinal mucin MUC2 is expressed in every intestinal metaplasia, which is a lesion that appears during the process of gastric carcinogenesis in the context of *H. pylori *infected individuals.

## Conclusion

Although *H. pylori *has been classified as a class I carcinogen by the IARC [4] the mechanism of gastric carcinogenesis remains poorly understood. In pre-neoplastic gastric mucosa, the expression of all the three mucin antigens did not appear to correlate with the severity of inflammation and *H. pylori *status. Results from the present study may suggest that *H. pylori *infection acts as an initiator of this carcinogenesis cascade by inducing inflammation and, in some cases, the subsequent metaplastic change. Intestinal metaplasia is accompanied by the induction of expression MUC2 and decreased expression of MUC5AC and MUC6. These alterations may favor the development of gastric carcinoma. The present study suggests that MUC2 is a marker of intestinal metaplasia and may be used for the early detection of this lesion in *H. pylori *infected pre-neoplastic human gastric epithelium.

## Materials and methods

### Tissue samples

Fifty nine gastric biopsy specimens and 3 ml of blood were obtained from Stanley Medical College and Hospital, Chennai. All biopsy tissue samples were collected after informed consent. This study was carried out after obtaining clearance from the ethical board of the hospital. All biopsy specimens were fixed in 10% buffered formalin and routinely embedded in paraffin wax. 4 μm thin serial sections were cut and used for immunohistochemistry. *H. pylori *infection was confirmed by RUT and ELISA [43]. Hematoxylin and Eosin stained sections were used to classify gastritis graded according to revised Sydney classification [44], gastric tumors according to the classification of Lauren [45] and WHO classification [46]. We studied 7 cases of normal Human gastric epithelium (7 biopsies), 26 cases of Atrophic gastritis (26 biopsies), 21 cases of Intestinal metaplasia (21 biopsies), 17 cases of dysplasia (17 biopsies) and 36 cases of carcinoma in which 21 cases early carcinoma and 15 cases advanced carcinoma (16 biopsies and 20 surgical specimens).

### Monoclonal antibodies

A panel of three monoclonal antibodies was used to determine the expression of mucins (MUC2, MUC5AC and MUC6). The specificities, references and dilutions of the antibodies are listed in Table 1.

### Immunohistochemistry

Sections from normal, pre-neoplastic and neoplastic gastric tissues were immuno stained according to Reis *et al *[47] with slight modifications. Sections designed for neuraminidase treatment were washed twice in phosphate buffered saline (PBS), pH 7.4 and incubated with neuraminidase from *clostridium perfringens Type VI *(Sigma; St Louis, MO) diluted in 0.1 M Na-acetate buffer, pH 5.5 to a final concentration of 0.1 U/ml. The incubation was carried out for 2 h at 37°C and was followed by three washings in ice-cold PBS. Sections were treated with 0.5% H_2_O_2 _in methanol for 30 min, followed by 20 min of incubation with rabbit non-immune serum. Sections were rinsed and incubated with primary antibody (Table 1), over night at 4°C. The sections were rinsed and incubated with HRP conjucated rabbit anti-mouse secondary antibody (1:100 dilution) for 1 h followed by rinsing with PBS and developing with 0.05% 3,3'-diaminobenzidine tetrahydrochloride (DAB) (SRL, Mumbai, India) freshly prepared in PBS containing 0.1% H_2_O_2_. Sections were counter stained with hematoxylin, dehydrated, and mounted. All series including positive and negative controls were performed by substitution of the primary mAbs with immunoglobulins of the same class and at the same concentration.

### Scoring of mucins expression in *H. pylori *infected pre-neoplastic and neoplastic human gastric tissues

A semi quantitative approach was used to score the immunostaining of human gastric tissues. The slides were examined under light microscope, every 100 cells were scored in five different fields and the results were expressed as mean percentage. (-), negative; +, few positive cells (<25%); ++ well-defined areas with positive cells (25–50%); +++, extensive areas with positive cells (50–75%); ++++, most cells stained (>75%).

### Statistics

Data analysis was performed using statistical software package SPSS 10.0 for windows. The Chi-Square test or Fisher's exact test was used to analyze differences in frequencies. Spearman's correlation test was used to analyze correlation between parameters. P < 0.05 was considered statistically significant.

## Abbreviations

DAB – 3,3'-diaminobenzidine tetra hydrochloride; ELISA – Enzyme-linked immunosorbent assay; HRP – Horseradish peroxidase; IARC – International Agency for Research on Cancer; IM – Intestinal metaplasia; mAbs – Monoclonal antibodies; PBS – Phosphate buffered saline; WHO – World Health Organization; RUT – Rapid Urea Test.

## Authors' contributions

SDB carried out all experiments along with the designing and coordination of HD. VJ provided patients data and specimens. HD reviewed the immunohistochemical results and ND, CAR provided suggestion for the finalization of this paper. All authors read and approved the final manuscript.
